# Genetically determined serum bilirubin level and the risk of heart failure: A mendelian randomization study

**DOI:** 10.3389/fgene.2023.1067146

**Published:** 2023-01-13

**Authors:** Bo Guan, Mingyan Yang, Xing Shen, Yemei Wang, Yutong Liu, Ruihan Liu, Shijun Li, Jian Cao

**Affiliations:** ^1^ Medical School of Chinese PLA General Hospital, Beijing, China; ^2^ Department of Experimental Hematology and Biochemistry, Beijing Key Laboratory for Radiobiology, Beijing Institute of Radiation Medicine, Beijing, China; ^3^ Jinzhou Medical University, Jinzhou, China; ^4^ Geriatric Cardiology Department of the Second Medical Center and National Clinical Research Center for Geriatric Diseases, Chinese PLA General Hospital, Beijing, China

**Keywords:** bilirubin, heart failure, mendelian randomization, causal effect, Antioxidants

## Abstract

**Background:** The association between serum bilirubin level and heart failure (HF) was controversial in previous observational studies and the causal effects of bilirubin on HF have not been investigated. Here, we conducted a Mendelian randomization (MR) study to investigate the associations between genetically determined bilirubin level and HF.

**Methods:** Summary data on the association of single nucleotide polymorphisms (SNPs) with serum bilirubin levels were obtained from genome-wide association study (GWAS) for individuals of European descent and East Asian descent separately. Statistical data for gene-HF associations were extracted from three databases: the HERMES Consortium (47,309 cases and 930,014 controls), FinnGen study (30,098 cases and 229,612 controls) for European population and Biobank Japan (2,820 HF cases and 192,383 controls) for East Asian population. We applied a two-sample Mendelian randomization framework to investigate the causal association between serum bilirubin and HF.

**Results:** Findings from our MR analyses showed that genetically determined serum bilirubin levels were not causally associated with HF risk in either European or East Asian population (odds ratio [OR] = 1.01 and 95% confidence interval [CI] = .97–1.05 for HERMES Consortium; OR = 1.01 and 95% CI = .98–1.04 for FinnGen Study; OR = .82, 95% CI: .61–1.10 for Biobank Japan). These results remained unchanged using different Mendelian randomization methods and in sensitivity analyses.

**Conclusion:** Our study did not find any evidence to support a causal association between serum bilirubin and HF.

## Introduction

Heart failure (HF), as the end-stage of all sorts of cardiac disorders, has become a serious public health concern afflicting more than eight million of people in China and 64 million of patients worldwide (Guo et al., 2016; McDonagh et al., 2021). Due to the progress made in alleviating ischemic cardiomyopathy, growing prevalence in comorbidities and aging, the prevalence of HF has been constantly climbing (Ponikowski et al., 2016; Castiglione et al., 2022). Overwhelmed oxidative stress has been recognized as the common pathological mechanism underlying the development of HF (Lambeth, 2004; Luo et al., 2021; Pigazzani et al., 2022). Increased oxidative stress could result in myocardial growth abnormity, extracellular matrix remodeling and cardiac energy metabolism disturbance (Senoner and Dichtl, 2019; Rotariu et al., 2022; Singh et al., 2022). Currently, strategies aiming at alleviating the oxidative stress have become heated topics in treating HF (Wang et al., 2021; Pigazzani et al., 2022).

Serum bilirubin is derived from heme catabolism within aging erythrocyte and is regarded as an important endogenous antioxidant (Schwertner and Vítek, 2008; Vítek, 2012; Kunutsor et al., 2017). It was reported that moderately elevated serum bilirubin concentration could exert cellular protective effects on oxidative stress-related cardiovascular disorders (Sedlak and Snyder, 2004; Gazzin et al., 2016). An inverse association was found between the circulating bilirubin concentration and the incidence of coronary heart disease, hypertension, and stroke (Kunutsor et al., 2015; Choi et al., 2020). In lieu of the unignorable involvement of oxidative stress in various cardiovascular diseases, bilirubin may play an important role in HF (Singh et al., 2020; Lamina and Kronenberg, 2021). Previous observational studies reported significant association between bilirubin level and HF risk (Adamson et al., 2022). However, the circulating bilirubin level undergoes constant fluctuation and is easily affected by venous pressure level and hepatic function, especially in HF patients (Moller and Bernardi, 2013). Therefore, whether bilirubin level simply mirrors the cardiogenic hepatic implications or has causal effects on HF has not been determined.

Mendelian randomization (MR) is a form of analysis that makes use of genetic variants as instrumental variables (IVs) to estimate effects of risk factors on outcomes (Davey Smith and Hemani, 2014; Davies et al., 2018). MR analysis takes advantage of the naturally occurring random allocation of alleles at conception and is recognized to overcome the limitations of residual confounding and reverse causation in conventional observational studies (Sekula et al., 2016). With the development of genome-wide association study (GWAS), MR becomes highly suited to investigate the etiological roles of conventional risk factors in HF. Here, we conduct a two-sample MR analysis to investigate the association between genetically bilirubin level and HF in both European and East Asian population.

## Methods

### Study design and data source

**FIGURE 1:**
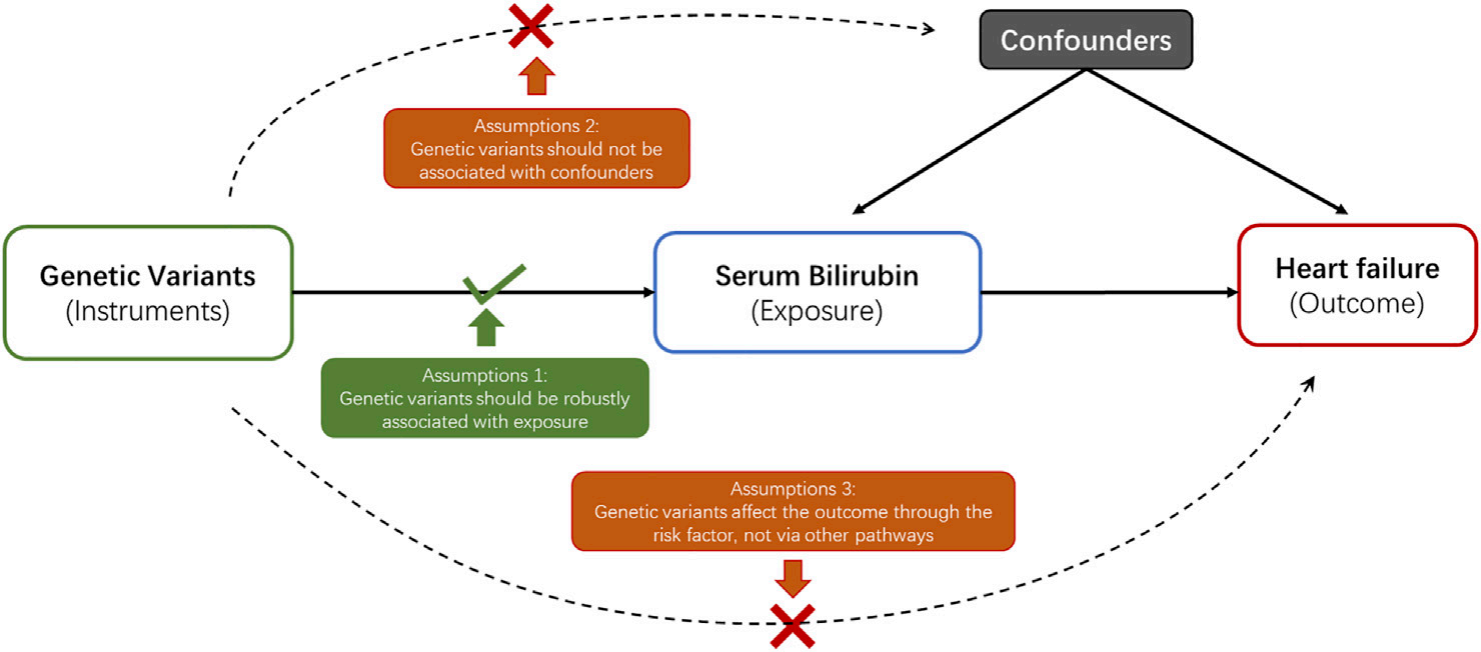
Three key assumptions underlying the Mendelian Randomization study design.

In current study, a 2-sample MR analysis was applied to assess the association between genetically determined serum bilirubin and HF in European population and East Asian population separately (Table 1). MR should abide by three principal assumptions (Burgess et al., 2015). First, the selected genetic instruments should be robustly associated with the exposure. Second, the association between these genetic instruments and the outcome should be exclusively through the exposure. Third, these genetic instruments should be independent of other potential cofounders (Figure 1).

**TABLE 1:** Detailed information for studies and datasets used for MR analysis.

Population	Contribution	Data source	Sample size	Number of SNPs
European	Exposure (Bilirubin)	United Kingdom Biobank	317,639	9,444,561
	Outcome (HF)	HERMES	977,323	7,773,021
		FinnGen	218,208	16,380,447
East Asian	Exposure (Bilirubin)	KoGES	25,406	∼830,000
	Outcome (HF)	Biobank Japan	212,453	8,885,805

MR, mendelian randomization; SNP, single nucleotide polymorphism; CI, confidence interval; HF, heart failure.

### Selection of genetic instruments

Candidate genetic instruments for total serum bilirubin levels were selected from United Kingdom (UK) Biobank and Korean Genome and Epidemiology Study (KoGES) for European and East Asian population respectively (Choi et al., 2020; Seyed Khoei et al., 2020). UK Biobank is a prospective cohort with genetic data collected on more than 500,000 individuals from 2006 to 2010 (Sinnott-Armstrong et al., 2021). Details concerning UK Biobank cohort can be obtained from online (https://www.ukbio bank. ac.uk). KoGES is a community-based cohort study recruited about 210,000 participants from 2005 to 2014 (Kim and Han, 2017). Among these participants, 25,406 were selected with both genetic data and serum bilirubin levels. Further detail information concerning KoGES can be found in previously published study (Kim and Han, 2017).

The single nucleotide polymorphisms (SNPs) reached genome-wide significance (*p* < 5*10^−8^ for serum bilirubin level were selected as instrumental variables. Independent SNPs were identified by linkage disequilibrium clumped using an *R* ^*2*^ threshold <.001. Palindromic SNPs were further removed to ensure that the effects of the SNPs on the exposure corresponded to the same allele as their effects on HF.

The strength of associations between the genetic instruments and bilirubin levels was reflected by F-statistic, which was calculated by formula previously described (Burgess and Thompson, 2011). To minimize potential weak instrument bias, SNPs with F-statistic more than 10 were considered sufficient to perform MR analysis and were included in current study. To ensure that the genetic variants used to proxy for bilirubin levels are valid genetic proxies, we performed a MR analysis on stroke as a positive control (Supplementary Table S1).

### Outcome data sets

Summary statistics for outcome data extraction in European population were from the published GWAS performed by the Heart Failure Molecular Epidemiology for Therapeutic Targets (HERMES) Consortium and the FinnGen study. The HERMES consortium enrolled 47,309 cases and 930,014 controls from 26 cohorts (Shah et al., 2020). HF cases were extracted based on the clinical diagnostic criteria regardless cause of disease with no specific inclusion criteria for left ventricular (LV) ejection fraction. Details of participants selection can be found elsewhere (Shah et al., 2020).

The FinnGen study is an ongoing nationwide GWAS launched in 2017. This study included genetic data from Finnish biobanks and health record data from Finnish health registries. Details on it including participating biobanks, genotyping, and data analysis can be found on official website (https://www.finng en. fi/en). The latest data, which included 30,098 reported heart failure and 229,612 controls of the FinnGen study (Release 6) were used in current analysis.

For East Asian population, GWAS of BioBank Japan (BBJ) was used for outcome data extraction. BBJ collected genetic and clinical information from more than 201,800 participants from April 2003 to February 2008 and follow-up for 47 target diseases (Ishigaki et al., 2020). Detailed information can be acquired from official website (https://biobankjp.org/en/index.htmlcv). Outcome information of our current analysis were from 2,820 HF cases and 192,383 controls.

The summary genetic association data for HERMES consortium and FinnGen Study are presented in Supplementary Table S2 and Supplementary Table S3 respectively, and the data for BioBank Japan are reported in Supplementary Table S4. The sample overlap between data sources of exposures and outcomes is shown in Supplementary Table S5.

### Statistical analysis

For our primary MR analysis, we applied the inverse-variance-weighted (IVW) regression analysis. Specially, the effect on an exposure on an outcome is estimated as the ratio (Wald estimate) of the SNP-outcome association and the SNP-exposure association (Helte et al., 2019). The IVW method assumes the absence of invalid genetic instruments. Cochran’s *Q* statistic was used to test the heterogeneity among the estimated Wald ratios from different genetic variants. To examine if there was violation of MR assumptions due to directional pleiotropy, MR-Egger regression analysis was performed, with the intercept of MR-Egger to estimate the average pleiotropic effect across the genetic variants (Burgess and Thompson, 2017). The weighted median method was used to provide a reliable effect estimate if at least one-half of the instrumental variables were valid (Burgess et al., 2017). Furthermore, Mendelian Randomization Pleiotropy Residual Sum and Outlier (MR-PRESSO) was performed to detect and correct for horizontal pleiotropy through removing outliers (Verbanck et al., 2018). In addition, a leave-one-out sensitivity analysis was conducted to determine whether the results were affected by a single SNP.

All results are presented as odds ratios (ORs) and corresponding 95% confidence intervals (CIs) of the outcomes with per predicted increase in serum bilirubin level. Statistically significant is identified only a two-sided *p*-value was less than .05. All the analyses were carried out with the TwoSampleMR and MR-PRESSO packages with R version 4.0.2.

## Results

### Causal association of bilirubin with HF in european population

**FIGURE 2:**
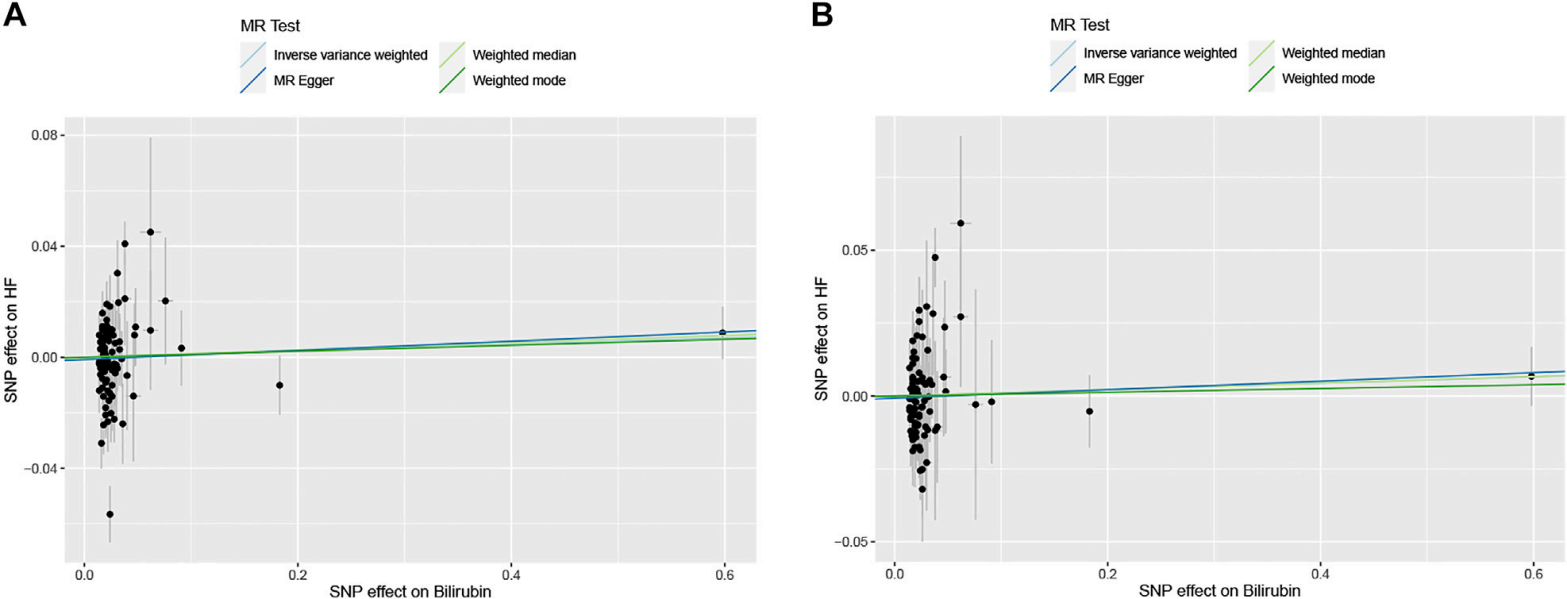
The scatter plots of serum bilirubin-associated single nucleotide polymorphisms (SNPs) effects on heart failure (HF) in HERMES Consortium **(A)** and FinnGen Study **(B)**. The 95% CI for the effect size on HF is shown as vertical lines, while the 95% CI for the effect size on bilirubin level is shown as horizontal lines. The slope of fitted lines represents the estimated Mendelian randomization (MR) effect per method.

The results of association between genetically determined bilirubin level and the risk of HF in HERMES Consortium and FinnGen Study were presented in Table 2 and Figure 2. Genetic predisposition to elevated bilirubin level was not associated with HF risk in both cohort by performing IVW method (OR = 1.01 and 95% CI: .98–1.04 in HERMES Consortium; OR = 1.01, 95% CI:0.98–1.05 in FinnGen Study). Sensitivity analyses including weighted-median, MR-Egger, and MR-PRESSO did not find substantial change with IVW analysis (all *p* > .05).

**TABLE 2:** Association between genetically determined bilirubin and heart failure in European Population.

Methods	Or (95% CI)	IVs (SNPs)	*p*-value	Q-statistics	*P*h
**For HERMES consortium**					
IVW	1.01 (.97–1.05)	85	.569	173.24	<.001
Weighted median	1.01 (.98–1.04)	85	.399		
MR-Egger	1.02 (.97–1.06)	85	.472		
Intercept^a^	−.001 (.002)		.624		
MR-PRESSO	1.01 (.98–1.04)	83	.582		
**For FinnGen study**					
IVW	1.01 (.98–1.05)	83	.536	104.48	.048
Weighted median	1.01 (.98–1.05)	83	.509		
MR-Egger	1.01 (.98–1.05)	83	.458		
Intercept^a^	−.001 (.002)		.655		
MR-PRESSO	1.01 (.98–1.05)	83	.537		

IVW, inverse-variance weighted; OR: odds ratio; CI: confidence interval; *P*h, *P* for heterogeneity.

^a^ Intercept is presented as β coefficients with SEs.

The MR-Egger intercept estimate did not detect significant directional pleiotropy. MR-PRESSO analysis found two outliers (rs2519093 and rs3184504) and the results remained unchanged after excluding the outliers. Significant heterogeneity was observed among individual SNPs for both cohorts. The result of leave-one-out sensitivity analysis showed the association between bilirubin and HF was not substantially driven by any individual SNP (Supplementary Figure S1).

### Causal relationship between bilirubin and HF in East Asian population

**FIGURE 3:**
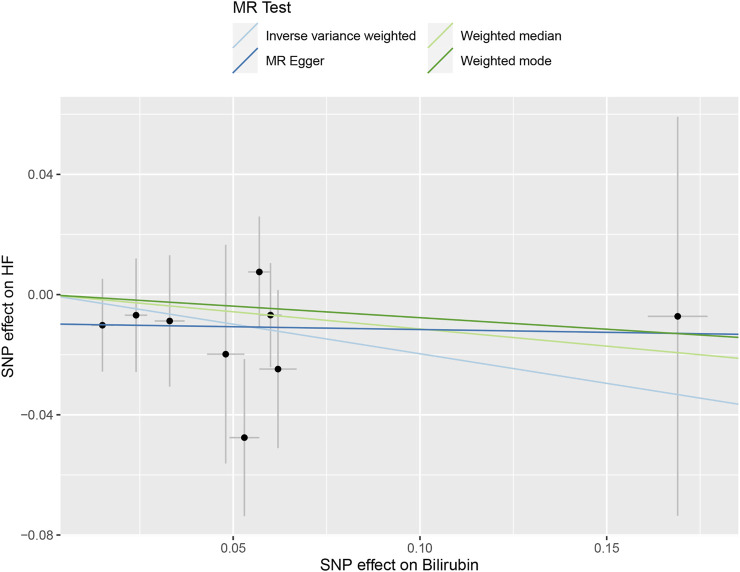
The scatter plot of serum bilirubin-associated single nucleotide polymorphisms (SNPs) effects on heart failure (HF) in the BioBank Japan cohort. The 95% CI for the effect size on HF is shown as vertical lines, while the 95% CI for the effect size on bilirubin level is shown as horizontal lines. The slope of fitted lines represents the estimated Mendelian randomization (MR) effect per method.

Table 3 and Figure 3 depicted the association between genetically determined serum bilirubin and HF risk in BioBank Japan Project. No significant causal effects of genetically determined bilirubin level on HF was observed in East Asian population by IVW method (OR = .82, 95% CI: .61–1.10). The results remained consistent in weighted-median, MR-Egger, and MR-PRESSO methods.

**TABLE 3:** Association between genetically determined bilirubin and heart failure in East Asian Population.

Methods	Or (95% CI)	IVs (SNPs)	*p*-value	Q-statistics	*P*h
IVW	.82 (.61–1.10)	9	.190	3.84	.871
Weighted median	.89 (.61–1.31)	9	.559		
MR-Egger	.98 (.53–1.81)	9	.954		
Intercept^a^	−.010 (.010)		.380		
MR-PRESSO	.82 (.65–1.04)	9	.095		

^a^ Intercept is presented as β coefficients with SEs.

The intercept estimates of MR-Egger method indicated no significant directional pleiotropy and MR-PRESSO method also did not find any potential outliers. Likewise, no heterogeneity was observed among individual SNPs of bilirubin for HF. In addition, leaving out each SNP did not cause substantial change to original results (Supplementary Figure S2).

The associations between direct and indirect bilirubin level with HF were also tested. There was only one SNP for direct bilirubin and one for indirect bilirubin. No significant association was observed either for direct bilirubin or indirect bilirubin with HF (Supplementary Table S6).

## Discussion

In the current study, we investigated the etiological role of bilirubin in HF by conducting a two-sample MR analysis. Our results did not find any significant association between genetically determined bilirubin level and HF risk in either European or East Asian population, indicating no causal effects of bilirubin on HF.

Bilirubin, as one kind of important antioxidants in plasma, was reported to exert about one-quarter of the total integral radical scavenging activities (Gopinathan et al., 1994). Evidence from conventional observational studies found that elevated bilirubin level was associated with decreased risk of several cardiovascular diseases. Wang et al., reported that serum bilirubin level was inversely correlated with hypertension risk (Wang and Bautista, 2015). Similarly, Lin et al., found that patients with gene prone to elevated bilirubin level showed a lower chance of coronary heart disease (Lin et al., 2006). Furthermore, Kimm et al., also found that low bilirubin level could serve as an independent risk factor of stroke (Kimm et al., 2009). However, the association between bilirubin and HF was much more complicated. In CHARM and PARADIGM-HF trial, total bilirubin levels were reported to elevate at baseline in 13% of all HF patients and 11.6% of patients with HF with reduced ejection fraction (HFrEF). Similarly, the EVREST and CHARM study both confirmed elevated bilirubin as a strong predictor of cardiovascular death in HF (Allen et al., 2009; Suzuki et al., 2020). However, some studies indicated that high bilirubin level was not associated with long-term cardiac remodeling and dysfunction as well as sudden cardiac death among HF patients (Wu et al., 2013). It should be also noted that serum bilirubin level is easily influenced by liver function and venous pressure, both of which were altered in most HF patients. Therefore, whether the change of serum bilirubin is a reflection of their involvement in disease pathogenesis or an epiphenomenon of HF induced hepatic injury remains ambiguous.

In recent years, MR research that uses genetic variants inherited randomly from parents has been acknowledged as a reliable method to infer a causal relationship between exposures and outcomes. With the aid of large-scale GWAS datasets, two-sample MR analysis has been widely used to examine the etiological roles of risk factors that reported in conventional observational studies in the development of various cardiovascular diseases (Lamina and Kronenberg, 2021). Similar to our study, previous MR analysis reported non-significant causal associations between cystatin C, galectin-3 and C-reactive protein with HF (van der Laan et al., 2016; Li et al., 2021; Wang et al., 2022), suggesting that these factors may function as bystanders instead of contributors to HF. While most of the previous MR analyses were conducted in European population, our study conducted a separate analysis by utilizing datasets in East Asian to limit the confounding effect of genetic background, which may improve the generalizability of our results.

Previous MR studies have suggested the association between genetically-determined bilirubin level and life-long atherosclerotic cardiovascular diseases (ASCVD), including coronary heart diseases and stroke (Lin et al., 2006; Kunutsor et al., 2017; Lee et al., 2017; Choi et al., 2020). However, the non-significant causal effects of bilirubin were detected on several cardiovascular diseases as well as HF in our current study (Hou et al., 2021). The divergent pathophysiological mechanisms underlying ASCVD and HF may partially explain the discrepancy. Evidence from preclinical studies revealed that bilirubin mainly exerted cardiovascular protection through protecting the endothelium from oxidative damage, maintaining normal flow-mediated vasodilation and inhibiting cholesterol oxidation (Erdogan et al., 2006; Yoshino et al., 2011; Maruhashi et al., 2019), which are major pathophysiological changes related to vascular dysfunction and damage, while no clear evidence showed that bilirubin has direct effects on pathological cardiac hypertrophy, matrix reorganization and cardiomyocyte apoptosis, which are cardinal pathophysiological changes in HF.

Since HF is a condition with heterogenous pathogenesis, the finding from our MR analysis should be treated with caution when extrapolated into the specific subtype of HF. Currently, HF can be classified into three subtypes based on LVEF level, including HF with reduced ejection fraction (HFrEF), HF with mid-range ejection fraction (HFmrEF), and HF with preserved ejection fraction (HFpEF) according to the ESC guidelines, and previous observational studies identified divergent risk factor profiles for these HF subtypes (Dunlay et al., 2017; McDonagh et al., 2021). Moreover, recent genetic studies found pronounced differences in the genetic architectures of HFrEF and HFpEF (Bielecka-Dabrowa et al., 2016; Joseph et al., 2022). For example, a recent large GWAS by Joseph et al. found 13 loci associated with HFrEF, but only one associated with HFpEF at genome-wide significance despite a robust sample size, indicating that HFpEF is likely to be a collective syndrome representing several different pathophysiological entities (Joseph et al., 2022). Therefore, the causal factors identified from MR analysis by combing all kinds of HF as an outcome may not play etiological roles in HFpEF. Future studies to address the genetic variants of type-specified HF and their influences on disease pathogenesis are warranted.

It should also be noted that the causal association between bilirubin and HF may vary in different populations. Previous epidemiological data reported that the population in Asia had higher prevalence of HF compared to that in Europe, both in adults and in the elderly (Lam, 2015; McDonagh et al., 2021), which may be resulted from the discrepancies in lifestyle pattern, environmental contributor, as well as genetic architecture. Similarly, variance of bilirubin levels was observed in different ethnicities, which could be partially due to the heterogeneity of genetic determinants (Dai et al., 2013). Thus, across-ancestry analysis could provide more useful information on the association between bilirubin and HF. Considering this, our current MR analysis incorporated genetic information from the largest GWAS of different population, including European and East Asian, and acquired consistent null causal effects of bilirubin on HF, indicating no etiological role of bilirubin in HF. However, extrapolation of our results to other populations still needs caution and future large GWAS in non-European and non-Asian population are needed.

Though the causal association between bilirubin and HF was not supported from MR analysis, it would not devalue the role of bilirubin acting as a prognostic factor for HF, as it goes for the role of C-reactive protein in coronary heart disease and interleukin 1β in Alzheimer’s disease. Bilirubin was reported as an extremely dynamic variable with the potential to change collaterally with disease status, and Adamson et al., proposed that bilirubin level might be more reflective on atrial pressure comparing with BNP (Adamson et al., 2022). Therefore, considering the spurious involvement of bilirubin in HF formation, using bilirubin level in predicting HF prognosis or risk stratification might be more instructive.

A major strength of the present study is the two-sample MR design to reduce bias from confounding factors and reverse causality. In addition, our MR analysis was conducted in both European and East Asian population, which can avoid the chance of results due to genetic divergency. However, some limitations must be noted. First, it is hard to avoid influence of potential directional pleiotropy completely, though no evidence of pleiotropic effect was observed in MR-Egger intercept test. However, SNPs associated with known confounding traits were excluded in our sensitivity analyses. Second, the sample overlapping between exposure dataset for bilirubin and outcome dataset for HF from HERMES consortium may cause some bias to our conclusion. However, our additional analysis using outcome data from FinnGen study to avoid sample overlapping did not find any significant change. Third, due to the unavailability of individual data, we could not conduct analyses grouped by sub-phenotypes and clinical courses of HF. Given the disparate pathophysiological underpinnings of different types of HF, further research in this regard is warranted. Fourth, generalizability of our findings to other ethnicities may be limited. Future large GWAS in other population are warranted.

## Conclusion

Our MR analysis did not identify convincing evidence to support the causal relationship between serum bilirubin and HF in either European or East Asian population. Additional human and animal studies are needed to confirm our MR results.

## Data Availability

The original contributions presented in the study are included in the article/Supplementary Material, further inquiries can be directed to the corresponding authors.

## References

[B1] Adamson C. Cowan L. M. de Boer R. A. Diez M. Drozdz J. Dukat A. (2022). Liver tests and outcomes in heart failure with reduced ejection fraction: Findings from DAPA-HF. Eur. J. heart Fail. 24 (10), 1856–1868. 10.1002/ejhf.2649 36054568 PMC9805158

[B2] Allen L. A. Felker G. M. Pocock S. McMurray J. J. Pfeffer M. A. Swedberg K. (2009). Liver function abnormalities and outcome in patients with chronic heart failure: Data from the candesartan in heart failure: Assessment of reduction in mortality and morbidity (CHARM) program. Eur. J. heart Fail. 11, 170–177. 10.1093/eurjhf/hfn031 19168515 PMC2639422

[B3] Bielecka-Dabrowa A. Sakowicz A. Misztal M. von Haehling S. Ahmed A. Pietrucha T. (2016). Differences in biochemical and genetic biomarkers in patients with heart failure of various etiologies. Int. J. Cardiol. 221, 1073–1080. 10.1016/j.ijcard.2016.07.150 27448535

[B4] Burgess S. Bowden J. Fall T. Ingelsson E. Thompson S. G. (2017). Sensitivity analyses for robust causal inference from mendelian randomization analyses with multiple genetic variants. Epidemiol. Camb. Mass) 28, 30–42. 10.1097/EDE.0000000000000559 PMC5133381 27749700

[B5] Burgess S. Scott R. A. Timpson N. J. Davey Smith G. Thompson S. G. (2015). Using published data in mendelian randomization: A blueprint for efficient identification of causal risk factors. Eur. J. Epidemiol. 30, 543–552. 10.1007/s10654-015-0011-z 25773750 PMC4516908

[B6] Burgess S. Thompson S. G. (2011). Avoiding bias from weak instruments in Mendelian randomization studies. Int. J. Epidemiol. 40, 755–764. 10.1093/ije/dyr036 21414999

[B7] Burgess S. Thompson S. G. (2017). Interpreting findings from Mendelian randomization using the MR-Egger method. Eur. J. Epidemiol. 32, 377–389. 10.1007/s10654-017-0255-x 28527048 PMC5506233

[B8] Castiglione V. Aimo A. Vergaro G. Saccaro L. Passino C. Emdin M. (2022). Biomarkers for the diagnosis and management of heart failure. Heart fail. Rev. 27, 625–643. 10.1007/s10741-021-10105-w 33852110 PMC8898236

[B9] Choi Y. Lee S. J. Spiller W. Jung K. J. Lee J. Y. Kimm H. (2020). Causal associations between serum bilirubin levels and decreased stroke risk: A two-sample mendelian randomization study. Arteriosclerosis, thrombosis, Vasc. Biol. 40, 437–445. 10.1161/ATVBAHA.119.313055 PMC6975519 31801373

[B10] Dai X. Wu C. He Y. Gui L. Zhou L. Guo H. (2013). A genome-wide association study for serum bilirubin levels and gene-environment interaction in a Chinese population. Genet. Epidemiol. 37, 293–300. 10.1002/gepi.21711 23371916

[B11] Davey Smith G. Hemani G. (2014). Mendelian randomization: Genetic anchors for causal inference in epidemiological studies. Hum. Mol. Genet. 23, R89–R98. 10.1093/hmg/ddu328 25064373 PMC4170722

[B12] Davies N. M. Holmes M. V. Davey Smith G. (2018). Reading mendelian randomisation studies: A guide, glossary, and checklist for clinicians. BMJ Clin. Res. ed) 362, k601. 10.1136/bmj.k601 PMC6041728 30002074

[B13] Dunlay S. M. Roger V. L. Redfield M. M. (2017). Epidemiology of heart failure with preserved ejection fraction. Nat. Rev. Cardiol. 14, 591–602. 10.1038/nrcardio.2017.65 28492288

[B14] Erdogan D. Gullu H. Yildirim E. Tok D. Kirbas I. Ciftci O. (2006). Low serum bilirubin levels are independently and inversely related to impaired flow-mediated vasodilation and increased carotid intima-media thickness in both men and women. Atherosclerosis 184, 431–437. 10.1016/j.atherosclerosis.2005.05.011 15979081

[B15] Gazzin S. Vitek L. Watchko J. Shapiro S. M. Tiribelli C. (2016). A novel perspective on the biology of bilirubin in health and disease. Trends Mol. Med. 22, 758–768. 10.1016/j.molmed.2016.07.004 27515064

[B16] Gopinathan V. Miller N. J. Milner A. D. Rice-Evans C. A. (1994). Bilirubin and ascorbate antioxidant activity in neonatal plasma. FEBS Lett. 349, 197–200. 10.1016/0014-5793(94)00666-0 8050565

[B17] Guo L. Guo X. Chang Y. Yang J. Zhang L. Li T. (2016). Prevalence and risk factors of heart failure with preserved ejection fraction: A population-based study in northeast China. Int. J. Environ. Res. public health 13, 770. 10.3390/ijerph13080770 27483300 PMC4997456

[B18] Helte E. Åkesson A. Larsson S. C. (2019). Assessing causality in associations of serum calcium and magnesium levels with heart failure: A two-sample mendelian randomization study. Front. Genet. 10, 1069. 10.3389/fgene.2019.01069 31708976 PMC6819429

[B19] Hou L. Li H. Si S. Yu Y. Sun X. Liu X. (2021). Exploring the causal pathway from bilirubin to CVD and diabetes in the UK biobank cohort study: Observational findings and Mendelian randomization studies. Atherosclerosis 320, 112–121. 10.1016/j.atherosclerosis.2020.12.005 33485635

[B20] Ishigaki K. Akiyama M. Kanai M. Takahashi A. Kawakami E. Sugishita H. (2020). Large-scale genome-wide association study in a Japanese population identifies novel susceptibility loci across different diseases. Nat. Genet. 52, 669–679. 10.1038/s41588-020-0640-3 32514122 PMC7968075

[B21] Joseph J. Liu C. Hui Q. Aragam K. Wang Z. Charest B. (2022). Genetic architecture of heart failure with preserved versus reduced ejection fraction. Nat. Commun. 13, 7753. 10.1038/s41467-022-35323-0 36517512 PMC9751124

[B22] Kim Y. Han B. G. (2017). Cohort profile: The Korean genome and Epidemiology study (KoGES) consortium. Int. J. Epidemiol. 46, 1350. 10.1093/ije/dyx105 28938752 PMC5837323

[B23] Kimm H. Yun J. E. Jo J. Jee S. H. (2009). Low serum bilirubin level as an independent predictor of stroke incidence: A prospective study in Korean men and women. Stroke 40, 3422–3427. 10.1161/STROKEAHA.109.560649 19713538

[B24] Kunutsor S. K. Bakker S. J. Gansevoort R. T. Chowdhury R. Dullaart R. P. (2015). Circulating total bilirubin and risk of incident cardiovascular disease in the general population. Arteriosclerosis, thrombosis, Vasc. Biol. 35, 716–724. 10.1161/ATVBAHA.114.304929 25593130

[B25] Kunutsor S. K. Kieneker L. M. Burgess S. Bakker S. J. L. Dullaart R. P. F. (2017). Circulating total bilirubin and future risk of hypertension in the general population: The prevention of renal and vascular end-stage disease (PREVEND) prospective study and a mendelian randomization approach. J. Am. Heart Assoc. 6, e006503. 10.1161/JAHA.117.006503 29133521 PMC5721749

[B26] Lam C. S. P. (2015). Heart failure in southeast Asia: Facts and numbers. Esc. heart Fail. 2, 46–49. 10.1002/ehf2.12036 28834655 PMC6410537

[B27] Lambeth J. D. (2004). NOX enzymes and the biology of reactive oxygen. Nat. Rev. Immunol. 4, 181–189. 10.1038/nri1312 15039755

[B28] Lamina C. Kronenberg F. (2021). The causal association of bilirubin with cardiovascular disease: Are there still any questions? Atherosclerosis 320, 92–94. 10.1016/j.atherosclerosis.2021.01.020 33541708

[B29] Lee S. J. Jee Y. H. Jung K. J. Hong S. Shin E. S. Jee S. H. (2017). Bilirubin and stroke risk using a mendelian randomization design. Stroke 48, 1154–1160. 10.1161/STROKEAHA.116.015083 28389615

[B30] Li X. Peng S. Guan B. Chen S. Zhou G. Wei Y. (2021). Genetically determined inflammatory biomarkers and the risk of heart failure: A mendelian randomization study. Front. Cardiovasc. Med. 8, 734400. 10.3389/fcvm.2021.734400 34881299 PMC8645870

[B31] Lin J. P. O'Donnell C. J. Schwaiger J. P. Cupples L. A. Lingenhel A. Hunt S. C. (2006). Association between the UGT1A1*28 allele, bilirubin levels, and coronary heart disease in the Framingham Heart Study. Circulation 114, 1476–1481. 10.1161/CIRCULATIONAHA.106.633206 17000907

[B32] Luo J. le Cessie S. van Heemst D. Noordam R. (2021). Diet-derived circulating antioxidants and risk of coronary heart disease: A mendelian randomization study. J. Am. Coll. Cardiol. 77, 45–54. 10.1016/j.jacc.2020.10.048 33413940

[B33] Maruhashi T. Kihara Y. Higashi Y. (2019). Bilirubin and endothelial function. J. Atheroscler. thrombosis 26, 688–696. 10.5551/jat.RV17035 PMC6711845 31270300

[B34] McDonagh T. A. Metra M. Adamo M. Gardner R. S. Baumbach A. Bohm M. (2021). 2021 ESC Guidelines for the diagnosis and treatment of acute and chronic heart failure. Eur. Heart J. 42, 3599–3726. 10.1093/eurheartj/ehab368 34447992

[B35] Moller S. Bernardi M. (2013). Interactions of the heart and the liver. Eur. heart J. 34, 2804–2811. 10.1093/eurheartj/eht246 23853073

[B36] Pigazzani F. Gorni D. Dyar K. A. Pedrelli M. (2022). The prognostic value of derivatives-reactive oxygen metabolites (d-ROMs) for cardiovascular disease events and mortality. Antioxidants (Basel) 11, 1541. 10.3390/antiox11081541 36009260 PMC9405117

[B37] Ponikowski P. Voors A. A. Anker S. D. Bueno H. Cleland J. G. F. Coats A. J. S. (2016). 2016 ESC Guidelines for the diagnosis and treatment of acute and chronic heart failure: The Task Force for the diagnosis and treatment of acute and chronic heart failure of the European Society of Cardiology (ESC)Developed with the special contribution of the Heart Failure Association (HFA) of the ESC. Eur. heart J. 37, 2129–2200. 10.1093/eurheartj/ehw128 27206819

[B38] Rotariu D. Babes E. E. Tit D. M. Moisi M. Bustea C. Stoicescu M. (2022). Oxidative stress - complex pathological issues concerning the hallmark of cardiovascular and metabolic disorders. Biomed. Pharmacother. = Biomedecine Pharmacother. 152, 113238. 10.1016/j.biopha.2022.113238 35687909

[B39] Schwertner H. A. Vítek L. (2008). Gilbert syndrome, UGT1A1*28 allele, and cardiovascular disease risk: Possible protective effects and therapeutic applications of bilirubin. Atherosclerosis 198, 1–11. 10.1016/j.atherosclerosis.2008.01.001 18343383

[B40] Sedlak T. W. Snyder S. H. (2004). Bilirubin benefits: Cellular protection by a biliverdin reductase antioxidant cycle. Pediatrics 113, 1776–1782. 10.1542/peds.113.6.1776 15173506

[B41] Sekula P. Del Greco M. F. Pattaro C. Köttgen A. (2016). Mendelian randomization as an approach to assess causality using observational data. J. Am. Soc. Nephrol. JASN. 27, 3253–3265. 10.1681/ASN.2016010098 27486138 PMC5084898

[B42] Senoner T. Dichtl W. (2019). Oxidative stress in cardiovascular diseases: Still a therapeutic target? Nutrients 11, 2090. 10.3390/nu11092090 31487802 PMC6769522

[B43] Seyed Khoei N. Jenab M. Murphy N. Banbury B. L. Carreras-Torres R. Viallon V. (2020). Circulating bilirubin levels and risk of colorectal cancer: Serological and mendelian randomization analyses. BMC Med. 18, 229. 10.1186/s12916-020-01703-w 32878631 PMC7469292

[B44] Shah S. Henry A. Roselli C. Lin H. Sveinbjornsson G. Fatemifar G. (2020). Genome-wide association and Mendelian randomisation analysis provide insights into the pathogenesis of heart failure. Nat. Commun. 11, 163. 10.1038/s41467-019-13690-5 31919418 PMC6952380

[B45] Singh R. B. Fedacko J. Pella D. Fatima G. Elkilany G. Moshiri M. (2022). High exogenous antioxidant, restorative treatment (heart) for prevention of the six stages of heart failure: The heart diet. Antioxidants 11, 1464. 10.3390/antiox11081464 36009183 PMC9404840

[B46] Singh R. B. Komatsu T. Lee M. C. Watanabe S. Nwozo S. O. Kiyoi T. (2020). Effects of behavioral risk factors with reference to smoking on pathophysiology of cardiomyocyte dysfunction. Journal 12, 9–13.

[B47] Sinnott-Armstrong N. Tanigawa Y. Amar D. Mars N. Benner C. Aguirre M. (2021). Genetics of 35 blood and urine biomarkers in the UK Biobank. Nat. Genet. 53, 185–194. 10.1038/s41588-020-00757-z 33462484 PMC7867639

[B48] Suzuki K. Claggett B. Minamisawa M. Packer M. Zile M. R. Rouleau J. (2020). Liver function and prognosis, and influence of sacubitril/valsartan in patients with heart failure with reduced ejection fraction. Eur. J. heart Fail. 22, 1662–1671. 10.1002/ejhf.1853 32407608

[B49] van der Laan S. W. Fall T. Soumaré A. Teumer A. Sedaghat S. Baumert J. (2016). Cystatin C and cardiovascular disease: A mendelian randomization study. J. Am. Coll. Cardiol. 68, 934–945. 10.1016/j.jacc.2016.05.092 27561768 PMC5451109

[B50] Verbanck M. Chen C. Y. Neale B. (2018). Detection of widespread horizontal pleiotropy in causal relationships inferred from Mendelian randomization between complex traits and diseases. Nat. Genet. 50, 693–698. 10.1038/s41588-018-0099-7 29686387 PMC6083837

[B51] Vítek L. (2012). The role of bilirubin in diabetes, metabolic syndrome, and cardiovascular diseases. Front. Pharmacol. 3, 55. 10.3389/fphar.2012.00055 22493581 PMC3318228

[B52] Wang H. Jia Q. Shi J. Hu Y. (2021). Prognostic value of serum bilirubin in patients with heart failure: A protocol for a systematic review and meta-analysis. Medicine 100, e26180. 10.1097/MD.0000000000026180 34087882 PMC8183714

[B53] Wang L. Bautista L. E. (2015). Serum bilirubin and the risk of hypertension. Int. J. Epidemiol. 44, 142–152. 10.1093/ije/dyu242 25541554 PMC6597028

[B54] Wang X. Wang X. Zhu J. Liu Y. Zhuang L. Zhang Z. (2022). Exploring the causal effects of circulating ST2 and galectin-3 on heart failure risk: A mendelian randomization study. Front. Cardiovasc. Med. 9, 868749. 10.3389/fcvm.2022.868749 35479285 PMC9037587

[B55] Wu A. H. Levy W. C. Welch K. B. Neuberg G. W. O'Connor C. M. Carson P. E. (2013). Association between bilirubin and mode of death in severe systolic heart failure. Am. J. Cardiol. 111, 1192–1197. 10.1016/j.amjcard.2012.12.048 23351460

[B56] Yoshino S. Hamasaki S. Ishida S. Kataoka T. Yoshikawa A. Oketani N. (2011). Relationship between bilirubin concentration, coronary endothelial function, and inflammatory stress in overweight patients. J. Atheroscler. thrombosis 18, 403–412. 10.5551/jat.6346 21350306

