# Supplementary material for: Genetically determined serum bilirubin level and the risk of heart failure: A mendelian randomization study
Source: Front Genet. 2023 Jan 13;14:1067146. doi: 10.3389/fgene.2023.1067146 (PMC9881886; doi:10.3389/fgene.2023.1067146)
Supplement: Supplementary file 2 [file Table1.DOCX]

**Supplementary Table Legends**

**Supplementary Table 1**. Summary statistics for the SNPs associated with bilirubin and their association with HF in the HERMES consortium.

**Supplementary Table 2.** Summary statistics for the SNPs associated with bilirubin and their association with HF in the FinnGen study.

**Supplementary Table 3.** Summary statistics for the SNPs associated with bilirubin and their association with HF in the Biobank Japan cohort.

**Supplementary Table 4.** Sample overlap between data sources of exposures and outcomes.

**Supplementary Table 5.** Results for MR analysis on stroke as a positive control outcome

**Supplementary Table 6.** Association between genetically determined direct and indirect bilirubin with heart failure in East Asian Population.

**Supplementary Figure Legends**

**Figure S1.** MR leave-one-out sensitivity analyses for bilirubin with heart failure in (A) HERMES Consortium and (B) FinnGen Study.

**Figure S2.** MR leave-one-out sensitivity analysis for bilirubin with heart failure in the Biobank Japan cohort.
